# A study on the relationship between physical exercise and feelings of inferiority among college students: the chain mediating effect of social support and emotional regulation ability

**DOI:** 10.3389/fpsyg.2024.1521510

**Published:** 2025-01-09

**Authors:** Bo Peng, Weisong Chen, Hongshen Wang, Ting Yu, Mingmin Kong

**Affiliations:** ^1^Sports Training Academy, Chengdu Sport University, Chengdu, Sichuan, China; ^2^Jing Shan Primary School, Chongqing, China

**Keywords:** physical exercise, feelings of inferiority, social support, emotional regulation ability, college students, structural equation model, chain mediation, mental health

## Abstract

**Objective:**

This study aims to explore the impact of physical exercise on feelings of inferiority among college students, focusing on the mediating roles of social support and emotional regulation ability. The research investigates both direct and indirect pathways to understand how physical exercise enhances psychological resilience and mitigates negative self-perceptions.

**Methods:**

A sample of 2,036 college students from 15 provinces in China was surveyed using validated scales for physical exercise, feelings of inferiority, social support, and emotional regulation ability. Structural equation modeling (SEM) was employed to test hypothesized pathways, including direct effects and chain mediation effects. Gender invariance was assessed through multi-group analysis to ensure consistency across male and female participants.

**Results:**

Physical exercise significantly reduces feelings of inferiority, both directly and indirectly. Social support and emotional regulation ability independently mediate this relationship, with social support further enhancing emotional regulation in a sequential chain mediation effect. Gender invariance testing confirmed that these relationships are consistent for male and female students. Key findings indicate that physical exercise fosters social connections and strengthens emotional regulation, collectively promoting psychological resilience and reducing feelings of inferiority.

**Conclusion:**

The findings emphasize the critical role of physical exercise in enhancing mental health among college students through social and emotional pathways. Holistic intervention programs incorporating physical activities with social interaction and emotional skill-building can effectively alleviate feelings of inferiority. These insights provide actionable recommendations for educational institutions to promote mental health and foster inclusive, supportive campus environments.

## 1 Introduction

Physical exercise, as an effective means of promoting health, has a profound impact on the physical and mental well-being of college students, particularly in the area of mental health, where its benefits are widely recognized. For college students, regular physical activity not only enhances physical fitness and self-efficacy but also plays an important role in alleviating emotional stress and enhancing psychological resilience (Chen et al., 2023; Chen et al., 2023). Despite the proven benefits of physical exercise, many college students struggle to maintain it over time, potentially due to underlying psychological barriers. Among these, Feelings of Inferiority—a common form of negative self-perception—have a significant impact on the psychological development of college students (Chen et al., 2006). Feelings of Inferiority not only hinder self-identity and emotional stability but also affect various aspects of college students’ lives, including social interactions, academic performance, and life satisfaction (Li and Kong, 2010; Wang and Lin, 2016). Therefore, exploring whether physical exercise can effectively alleviate feelings of inferiority has important theoretical and practical implications for promoting the mental and physical health of college students.

Existing research suggests that physical exercise can help individuals gain positive self-experiences, enhance self-esteem, and thereby reduce feelings of inferiority to some extent (Qin and Yuan, 2019; McAuley et al., 1993). However, the psychological state of college students is influenced by multiple factors, making the underlying mechanisms potentially more complex than in the general population. Social support, as an essential psychological resource, refers to the emotional, informational, and material resources an individual receives from family, friends, or the social environment, which can play a crucial role in psychological coping (Liu and Huang, 2010). Studies show that strong social support not only alleviates psychological stress in college students but also enhances their motivation to participate in physical exercise, increasing persistence and thereby improving mental health and self-identity (Wang and Bu, 2023). For college students with feelings of inferiority, social support may play a key role in the psychological benefits of physical exercise, providing emotional support and encouragement, which can help alleviate feelings of inferiority more effectively (Yang et al., 2022; Miao et al., 2020).

Simultaneously, emotional regulation ability—a core capacity for managing stress and controlling emotions—is essential to the psychological development of college students (Xue and Zhao, 2018). Individuals with strong emotional regulation ability are typically better able to control emotional fluctuations and face life’s pressures and setbacks constructively (Du et al., 2015). During physical exercise, emotional regulation ability enables individuals to better cope with the negative emotions that may arise from physical fatigue or challenges, making it easier to experience the positive psychological benefits of exercise (Wang et al., 2023; Eckert et al., 2016).

For college students with feelings of inferiority, stronger emotional regulation ability may help them more effectively reduce negative emotions during physical exercise, thereby gradually enhancing self-identity (Lebowitz and Dovidio, 2015). Thus, emotional regulation ability not only strengthens psychological resilience in the context of physical exercise but may also alleviate feelings of inferiority by improving self-emotional management.

Based on this background, this study aims to explore how physical exercise positively impacts Feelings of Inferiority in college students from the dimensions of social support and emotional regulation ability. By analyzing the mechanisms of these psychological and social factors, the study seeks to reveal pathways through which physical exercise alleviates feelings of inferiority, providing scientific evidence and practical guidance for educational and psychological interventions. College students are in a critical stage of self-concept and social role formation, and understanding and supporting their psychological development can help reduce feelings of inferiority, improve overall quality of life, and enhance psychological adaptability. The findings of this study will provide empirical support for universities to design more effective mental health support programs and physical exercise initiatives, helping students achieve balance in their physical and mental development and promoting comprehensive enhancement of mental health and personal growth.

## 2 Literature review and research hypotheses

### 2.1 The direct effect of physical exercise on feelings of inferiority in college students

In recent years, the role of physical exercise in promoting mental health has gained increasing attention, particularly regarding its effectiveness in alleviating feelings of inferiority and enhancing self-identity and self-evaluation. Research has demonstrated that physical exercise not only improves physical condition but also has significant positive effects on psychological well-being (Cai et al., 2004; Li, 2024). This positive influence is particularly important for college students, who are in a critical phase of developmental growth. Feelings of inferiority, as a common form of negative self-perception, impact not only the mental health of college students but also their academic performance, social relationships, and life satisfaction in various ways (Li and Li, 2015). Therefore, studying the mechanisms by which physical exercise alleviates feelings of inferiority in college students holds important theoretical and practical value.

Physical exercise provides college students with opportunities for self-affirmation and self-improvement. Regular exercise not only helps improve physical appearance but also gradually increases satisfaction with body image. Body image is an important dimension of self-evaluation, and positive experiences in physical appearance and fitness gained through exercise enhance students’ self-recognition (Lu, 2000; Chen, 2007). Research indicates that regular physical exercise significantly improves college students’ physical appearance, posture, and strength, which boosts their confidence in both self-perception and in front of others. For students who experience feelings of inferiority due to appearance or physical ability, the physical changes resulting from exercise effectively reduce negative self-perceptions, thereby alleviating feelings of inferiority (Qiu et al., 2014). Beyond improvements in body image, the sense of achievement and control gained through physical exercise plays a critical role in enhancing self-identity. Setting and achieving exercise goals provides college students with a clear sense of growth, whether through improved athletic performance or the accomplishment of specific training objectives. This process allows students to experience achievement, which is a source of increased self-efficacy, fostering positive self-evaluation through continuous goal attainment (Xiao et al., 2024). Students who experience this sense of accomplishment through exercise tend to exhibit greater confidence and more positive self-identity. This achievement-based psychological feedback helps them reassess their self-worth, reducing Feelings of Inferiority triggered by failure or setbacks (Chen, 2012).

On the physiological level, physical exercise generates significant positive effects. Exercise promotes the release of “happiness hormones” such as endorphins, which induce feelings of pleasure and satisfaction (Li et al., 2022; Bernstein and McNally, 2017). This positive physiological feedback not only improves emotional state but also fosters a more positive self-identity. Increased feelings of pleasure and satisfaction help college students gradually build a positive self-view, alleviating the negative self-perceptions associated with negative emotions (Duncan et al., 2014). This positive physiological impact allows students to adopt a more optimistic view of themselves and the world, reducing negative psychological states associated with feelings of inferiority (Tao et al., 2023). Notably, the psychological stability developed through long-term physical exercise also plays a crucial role in reducing feelings of inferiority. Compared to those who participate sporadically, college students who consistently engage in physical exercise exhibit greater stability and positivity in self-evaluation (Xie, 2001). Establishing a regular exercise habit not only brings physical benefits but also cultivates a stronger sense of self-identity and self-esteem. Through sustained physical exercise, college students can approach academic or life setbacks with a more positive mindset rather than easily falling into self-denial. These enduring psychological benefits help college students develop a solid self-identity and psychological resilience during their growth, aiding in the reduction of Feelings of Inferiority (Qin and Yuan, 2019; Cheng and Liu, 2015). Additionally, the self-discipline cultivated through physical exercise plays an important role in enhancing students’ self-worth. Physical exercise requires consistent effort and patience, and by achieving self-discipline and goal attainment in exercise, students not only gain health benefits but also form a positive self-evaluation over time. Research shows that individuals with strong self-discipline tend to exhibit higher self-esteem and confidence, which provide crucial psychological support for alleviating Feelings of Inferiority (Li et al., 2018; Li et al., 2024). The self-discipline and persistence developed through physical exercise offer college students an effective pathway for establishing positive self-identity and help them approach life and academic challenges with increased confidence and resilience (Simard et al., 2023; Wang et al., 2024).

Based on this understanding, we propose the following hypothesis:

*Hypothesis 1 (H1*): Physical exercise has a direct negative effect on feelings of inferiority among college students, where higher levels of physical exercise are associated with lower levels of feelings of inferiority.

### 2.2 The mediating role of social support

In recent years, the critical role of social support in the field of mental health has gained wide recognition, particularly in reducing feelings of inferiority and enhancing self-identity. Social support is regarded as a core psychological resource that not only directly improves mental state but also plays a key role in self-acceptance and self-identity formation. Social Support typically includes emotional encouragement, behavioral reinforcement, and positive feedback from family, friends, and teams, which foster a sense of acceptance and recognition, thereby enhancing self-worth and self-identity (Liu and Huang, 2010). This positive feedback from Social Support helps individuals establish a stable self-evaluation system, reducing the feelings of inferiority that stem from negative self-perceptions (Huang et al., 2021; Wang, 2008). For college students, social support’s role in mental health is particularly crucial. College students, who are in a critical phase of self-awareness and social role development, are highly influenced by external evaluations and interpersonal relationships. Through participation in group activities, particularly Physical Exercise, college students can build positive social connections and emotional support networks (Shi and Zhou, 2017; Gong et al., 2020). Physical activities, especially team-based ones, provide an environment for interaction and collaboration, where students receive peer support, recognition, and encouragement. This sense of belonging not only reduces feelings of loneliness and isolation but also fosters positive self-perception, thereby alleviating feelings of inferiority (Liu, 2010). Studies have found that the sense of belonging obtained through group sports helps college students boost self-esteem through acceptance and recognition, leading them to develop a more positive self-view and reducing negative self-image perceptions (Ma and Liu, 2015).

Furthermore, social support has a buffering effect against psychological stress. A strong social support system enables individuals to manage stress and emotional fluctuations more effectively, especially when facing external criticism or personal setbacks (Miao et al., 2020). For college students, social support derived from physical exercise is especially vital. As college students are in a highly sensitive stage of self-awareness, they are more vulnerable to negative feedback, which can lead to negative self-perceptions (Ren et al., 2022). In such situations, peer support and team recognition in sports provide a strong emotional buffer, making them less prone to negative self-evaluation during setbacks, thereby reducing Feelings of Inferiority (Chen et al., 2016). The support network formed through Physical Exercise helps students gain emotional support and protection when facing challenges, building a more stable self-concept that resists self-negation in the face of adversity (Liu and Yin, 2010). Studies indicate that social support in physical exercise not only reduces feelings of inferiority in the short term but also has lasting effects. College students with high levels of social support exhibit stronger psychological resilience and a more positive self-evaluation system, helping them reinforce self-identity as they grow (Dong et al., 2018). The social support formed within physical exercise provides ongoing psychological support, enabling students to enhance self-identity and self-esteem, which lays a foundation for alleviating Feelings of Inferiority (Zhang and Xie, 2007). Additionally, social support in physical exercise can reduce feelings of inferiority by enhancing self-identity. Self-identity, an individual’s recognition of their role, value, and goals, provides a positive self-evaluation system (Xu and Dong, 2020; He et al., 2023). Peer recognition and team affirmation gained through collaboration help college students gradually understand their contributions and the importance of their roles, leading to greater self-confidence in the face of life and academic pressures (Liu, 2010). College students who experience strong social support in physical exercise typically have a stronger sense of self-identity and value in their roles, thereby alleviating negative emotions associated with feelings of inferiority.

Based on this understanding, we propose the following hypotheses:

*Hypothesis 2 (H2*): Physical exercise has a positive effect on social support among college students.

*Hypothesis 3 (H3*): Social support has a negative effect on feelings of inferiority among college students.

*Hypothesis 4 (H4*): Social support as a mediator between physical exercise and feelings of inferiority.

### 2.3 The mediating role of emotional regulation ability

The importance of emotional regulation ability in mental health has become increasingly prominent in recent years, particularly in coping with stress, managing emotions, and enhancing self-identity. Emotional regulation ability is defined as an individual’s capacity to effectively identify, manage, and regulate emotions in the face of emotional fluctuations. It serves as a foundation for psychological resilience and positive self-evaluation (Eisenberg et al., 2000; Wu and Lian, 2014). Individuals with strong emotional regulation ability are generally better equipped to maintain emotional stability when encountering setbacks or negative situations, thereby preventing emotional fluctuations from negatively impacting their self-identity and sustaining a positive self-view. This capacity not only helps reduce emotional stress but also enhances adaptability and confidence in coping with life and academic pressures (An and Pei, 2017). For college students, emotional regulation ability is especially critical in managing anxiety and coping with setbacks, helping them maintain a positive mindset during emotional fluctuations and thereby reduce negative self-evaluation (Han et al., 2024). Studies have shown that emotional regulation ability fosters a healthy psychological adaptation model, enabling students to view themselves and their environment more positively in the face of multiple pressures (Yao et al., 2022).

The positive impact of physical exercise on enhancing emotional regulation ability has been well-supported by empirical research. Physical activities not only improve physical fitness but also provide significant benefits in emotional management and stress coping. Forms of exercise such as aerobic activities, strength training, and team sports help release “feel-good” hormones like endorphins, providing positive emotional experiences that mitigate negative emotions and enhance emotional regulation ability (Li et al., 2022). Additionally, the persistent challenges and sense of achievement in sports offer college students a valuable opportunity to cultivate emotional stability. By regularly participating in Physical Exercise, college students gradually adapt to physical and technical challenges, fostering emotional stability and enabling them to approach stress with greater calmness and rationality (Hu, 2008; Guo and Zhang, 2000). Enhanced emotional regulation ability allows college students to quickly restore psychological balance when faced with setbacks or emotional fluctuations, thereby reducing the impact of negative emotions on self-evaluation (Gross, 2002). Moreover, emotional regulation ability indirectly helps reduce feelings of inferiority. College students with strong emotional regulation ability are more flexible in managing emotions, allowing them to quickly stabilize emotions in negative situations and prevent the accumulation of negative emotions that could lead to self-denial (Liu et al., 2020). For instance, when confronted with academic pressure or social setbacks, students with high emotional regulation ability tend to focus on problem-solving rather than self-criticism, maintaining a positive self-view. This capacity helps them reduce feelings of inferiority while displaying greater psychological resilience (Huang et al., 2016). Physical exercise cultivates emotional regulation Ability in college students, helping them avoid self-deprecation in times of emotional fluctuation and fostering a stable emotional response model. Specifically, the process of coping with setbacks and adjusting emotions in physical exercise encourages students to adopt a calm and rational attitude during emotional fluctuations, effectively reducing the likelihood of feelings of inferiority (Tao et al., 2023).

The role of emotional regulation ability in alleviating feelings of inferiority is also evident in enhanced psychological resilience. Individuals with strong emotional regulation ability can quickly recover positive emotions after experiencing stress, forming a positive emotional cycle that enables them to restore psychological balance more swiftly. During physical exercise, emotional regulation ability helps college students quickly regain their psychological state after setbacks, gradually establishing a positive emotional feedback mechanism. This allows them to view academic or life pressures as opportunities for self-improvement, rather than falling into feelings of inferiority (Huang et al., 2016). For example, through coping with setbacks in physical exercise, college students gradually learn to view failures as growth opportunities, building a positive self-perception system. Emotional regulation ability improves through the sustained practice of physical exercise, equipping college students with greater self-identity and confidence in their psychological growth (Wang, 2021). Additionally, the improvement of emotional regulation ability through physical exercise is reflected in long-term emotional stability and positive self-evaluation. Individuals who engage in regular physical activities tend to be more mature in emotional regulation, making them less likely to let short-term emotional fluctuations affect their self-identity and allowing them to maintain a positive self-image. Physical exercise helps college students develop adaptive coping strategies for emotional management, fostering strong self-esteem and self-acceptance, which gradually alleviates Feelings of Inferiority (Du and Luo, 2017). Enhanced emotional regulation ability through long-term exercise allows college students to restore psychological balance quickly during setbacks and gradually build confidence and self-recognition (Han et al., 2024).

Based on this understanding, we propose the following hypotheses:

*Hypothesis 5 (H5*): Physical exercise has a positive effect on emotional regulation ability among college students.

*Hypothesis 6 (H6*): Emotional regulation ability has a negative effect on feelings of inferiority among college students.

*Hypothesis 7 (H7*): Emotional regulation ability as a mediator between physical exercise and feelings of inferiority.

### 2.4 The chain mediating role of social support and emotional regulation ability

In recent years, the significant role of physical exercise in improving mental health has garnered considerable attention, particularly for its multi-layered positive mechanisms in providing social support and enhancing emotional regulation ability. For college students, physical exercise not only brings physical health benefits but also generates extensive positive effects on mental health, helping them cope with academic, life, and social pressures (Qin and Yuan, 2019). As a social activity, especially in team settings, physical exercise provides college students with frequent opportunities for social interactions, enabling them to establish a supportive emotional network within the group. Through teamwork, interaction, and competition, college students form new friendships and build emotional bonds, gaining a sense of belonging and emotional solace (Wang et al., 2010). This social support developed through physical exercise acts as an emotional buffer, reducing psychological burdens in the face of loneliness or alienation (Zhang and Wang, 2006).

Social support plays a critical role in emotion management and stress coping. Individuals with a stable social support network are generally better able to maintain emotional stability, especially when facing challenges or stress. Emotional encouragement and behavioral support help individuals develop stronger emotional regulation ability (Zhuang et al., 2021). The support obtained through physical exercise allows college students to be more adaptable in managing emotions, fostering resilience in emotional regulation. The support system in team sports provides students with a sense of psychological security, enabling them to rely on peer support and encouragement to cope with emotional fluctuations and become less sensitive to external negative evaluations (Widnall et al., 2021). When social support serves as a psychological resource, individuals can more calmly cope with negative emotions and stress, exhibiting greater emotional resilience and adaptability in the face of setbacks. Research suggests that social support not only helps college students develop emotional regulation ability but also promotes a balanced mental state under pressure (Gu et al., 2024).

The enhancement of emotional regulation ability is an additional psychological benefit resulting from the social support gained through physical exercise. With improved emotional regulation ability, college students can quickly regain emotional balance when facing negative emotions or stress, avoiding internalizing stress as negative self-perception. Emotional regulation ability helps students maintain a positive mindset during emotional fluctuations, preventing temporary emotional ups and downs from transforming into prolonged Feelings of Inferiority (Du et al., 2015). This ability plays a crucial role in emotional coping, allowing students to manage and adjust their mindset effectively when encountering negative emotions, gradually reducing tendencies toward self-criticism and self-devaluation. College students with strong emotional regulation ability tend to focus on problem-solving under pressure, rather than dwelling on negative emotions, forming a more positive self-perception system and reducing Feelings of Inferiority (An and Pei, 2017). The emotional regulation ability cultivated through physical exercise helps students establish a stable emotional response model, enabling them to remain calm and rational under stress, achieving emotional self-balance.

The positive influence of physical exercise on emotional regulation extends beyond short-term stress relief, promoting long-term emotional stability and positive self-identity. College students who participate in physical exercise regularly tend to exhibit higher levels of emotional regulation maturity, making them less likely to experience negative self-evaluation or loss of self-identity due to short-term emotional fluctuations. This emotional regulation maturity helps them develop effective coping strategies for negative emotions and enhances their sense of control over their emotions through repeated practice (Song, 2014). As emotional regulation ability improves, students can quickly regain psychological balance when facing setbacks, avoiding deepened feelings of inferiority due to emotional distress and building stable self-esteem and self-acceptance (Wang and Wang, 2013). This improvement in emotional regulation ability achieved through physical exercise fosters positive self-affirmation, equipping students with greater psychological resilience when confronting life challenges (Shi and Zhou, 2017).

Based on this understanding, we propose the following hypothesis:

*Hypothesis 8 (H8*): Physical exercise influences feelings of inferiority in college students through the chain mediating effects of social support and emotional regulation ability.

### 2.5 Construction of the integrated theoretical hypothesis model

**Figure 1:**
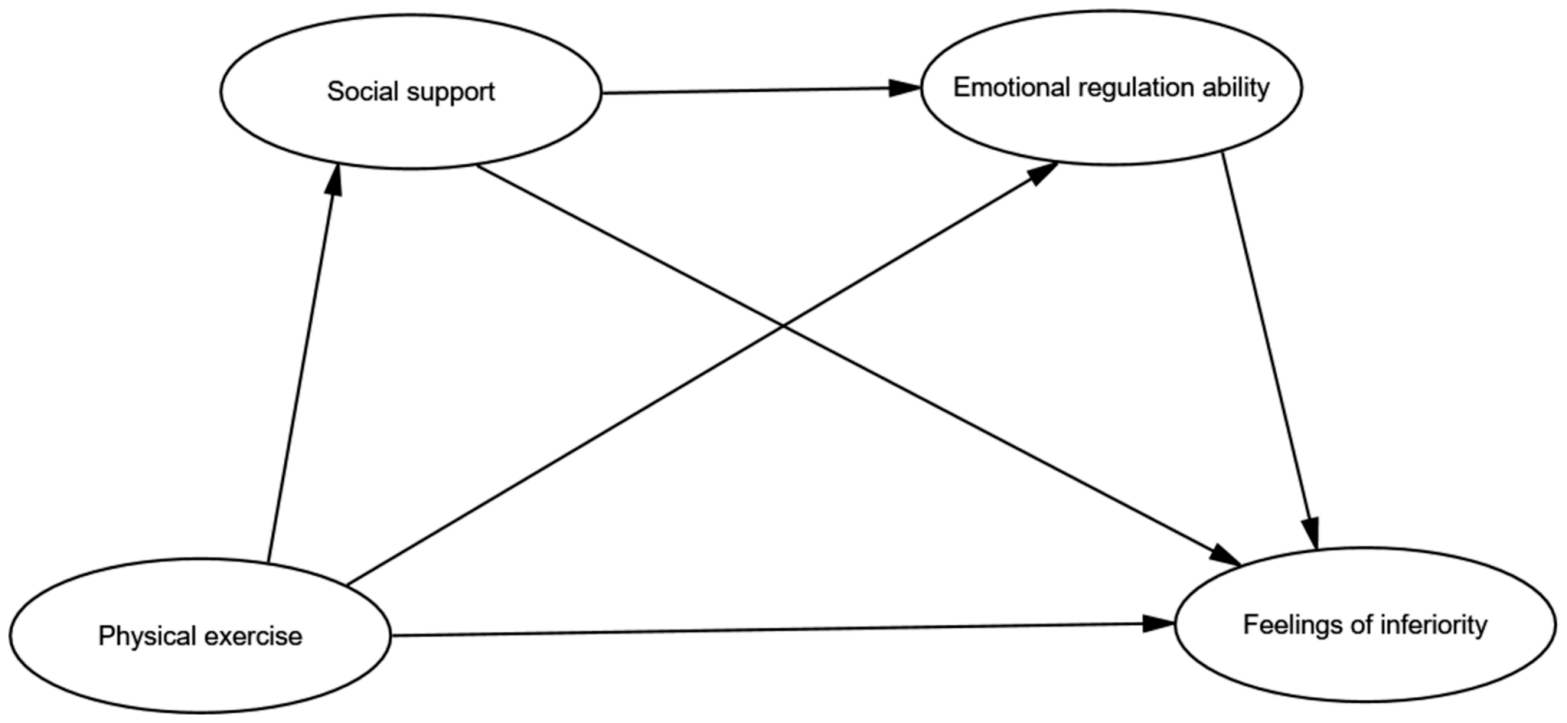
Theoretical model of the relationship between physical exercise and feelings of inferiority among college students: the chain mediating role of social support and emotional regulation ability.

Based on the literature review and relevant hypotheses, this study has developed a theoretical hypothesis model, as shown in Figure 1. This model aims to integrate existing research findings, optimize the relationships between hypotheses, and expand the theoretical framework for a more comprehensive understanding of the research topic.

## 3 Materials and methodology

### 3.1 Participants and data

#### 3.1.1 Sample size justification

To ensure the robustness and reliability of the research, we conducted a sample size estimation using G*Power software and general principles from sociological research.

We performed a power analysis for multiple regression using G*Power 3.1, involving four independent variables (physical exercise, social support, emotional regulation ability, and feelings of inferiority). Assuming a medium effect size (*f*^2^ = 0.15), a significance level of *α* = 0.05, and a desired statistical power of 1-*β* = 0.80, the analysis indicated that at least 90 participants are needed to detect a significant statistical effect.

In addition to the G*Power analysis, we also referenced widely accepted principles in sociological research, which suggest that the number of participants should be at least 10 to 15 times the number of questionnaire items. In this study, the physical exercise scale contains 3 items, the social support scale contains 12 items, the emotional regulation ability scale contains 14 items, and the feelings of inferiority scale contains 36 items, totaling 65 items. According to this principle, the minimum required sample size should be between 650 and 975 participants (65 items × 10 to 65 items × 15 participants).

By combining these two methods, we ensured that the sample size was adequate and exceeded the minimum requirements. The final sample comprised 2,036 university students, far exceeding both the G*Power minimum requirement and the empirical guideline threshold. Such a large sample not only enhances the statistical power of the study but also improves the generalizability and reliability across different population groups.

#### 3.1.2 Participant selection process

Participants were selected using a stratified random sampling technique to ensure the representation of different regions and population groups, thereby minimizing selection bias. The sampling framework included university students from 15 provinces and cities in China.

The randomization process consisted of multiple levels: first, the regions were stratified, and schools were randomly selected within these strata; then, students were randomly chosen to participate from the selected schools. This multilevel randomization ensured the representativeness of the student sample from different regions of China. The selection process included the following specific steps:

##### 3.1.2.1 Stratification by region

Based on the socioeconomic and cultural diversity of China, the country was divided into five geographical strata. Three provinces or cities were selected from each region to ensure balanced representation. Table 1 shows the specific regions and selected provinces.

**Table 1:** Stratification by region and province in the sampling frame.

Region	Province
Eastern China	Shanghai, Jiangsu, Shandong
Central China	Henan, Hubei, Hunan
Western China	Sichuan, Guizhou, Chongqing
Southern China	Guangdong, Guangxi, Hainan
Northern China	Beijing, Shaanxi, Liaoning

##### 3.1.2.2 Random selection of schools

In each geographical stratum, schools were selected using a random number generator. The number of schools chosen from each region was proportional to the student population in that area, ensuring balanced representation. For example, if a region accounts for 20% of the total university student population, then 20% of the schools will be selected from that region.

##### 3.1.2.3 Random selection of students

In each selected university, students were randomly chosen from a list that included first-year to fourth-year students using a random number generator. This process ensured that each student had an equal chance of being selected, further reducing selection bias.

##### 3.1.2.4 Exclusion of certain regions

Due to logistical constraints, geographical remoteness, and challenges in obtaining research permits, certain areas (such as Xinjiang, Tibet, and Taiwan) were excluded from the sampling framework. Xinjiang and Tibet presented complexities in travel and data collection due to their vast geographic areas, remote locations, and diverse ethnic compositions. Taiwan posed additional challenges due to difficulties in obtaining research permits and participant cooperation. While the exclusion of these regions may limit the generalizability of the research findings to these specific areas, the included regions still provide a broadly representative sample of the university student population in China.

#### 3.1.3 Data collection methods

Data collection was conducted through standardized procedures to ensure the validity and reliability of the data. The specific steps were as follows:

##### 3.1.3.1 Training of data collectors

Before distributing the questionnaires, all members of the research team underwent comprehensive training. This training covered the research objectives, the importance of random sampling, and standardized instructions for administering the questionnaires to ensure consistency in the process.

##### 3.1.3.2 Questionnaire distribution

Questionnaires were distributed on regular school days, supervised by members of the research team to ensure that students completed the questionnaires independently and without external interference. Once completed, the questionnaires were immediately collected to ensure timely and accurate data recovery.

##### 3.1.3.3 Confidentiality and informed consent

This study strictly adhered to the Declaration of Helsinki and relevant national and institutional guidelines. The research received ethical approval from the Ethics Committee of Chengdu Sports University. As all participants were adults, verbal consent was obtained from each participant prior to their involvement, ensuring compliance with ethical standards. Data analysis was conducted anonymously to protect participants’ privacy and maintain the confidentiality of the data.

#### 3.1.4 Data processing

The survey was conducted from April 1, 2024, to September 1, 2024, with a total of 2,200 questionnaires distributed. After excluding invalid questionnaires due to errors, omissions, and fixed responses, 2,036 valid questionnaires were retrieved, resulting in a valid response rate of 92.55%. Invalid questionnaires were filtered out based on predefined criteria (including incomplete responses and inconsistent answers) to ensure data quality and integrity. Table 2 presents the basic information of the survey participants, including the distribution of gender and grade. In terms of gender, the proportions of male and female participants are similar, with 1,011 males (49.66%) and 1,025 females (50.34%), indicating a relatively balanced overall distribution. Regarding grade levels, the distribution of participants across different grades is relatively even, with 506 freshmen (24.85%), 503 sophomores (24.71%), 501 juniors (24.61%), and 526 seniors (25.83%). The cumulative percentage data further demonstrates the comprehensiveness of the sample coverage, ensuring strong representativeness and reliability for the analysis.

**Table 2:** The sample information.

Basic information	Category	Frequency	Percentage	Cumulative percentage
Gender	Male	1,011	49.66%	49.66%
	Female	1,025	50.34%	100%
Grade	Freshman (Year 1)	506	24.85%	24.85%
	Sophomore (Year 2)	503	24.71%	49.56%
	Junior (Year 3)	501	24.61%	74.17%
	Senior (Year 4)	526	25.83%	100%

### 3.2 Measurement

Physical exercise was assessed using a scale developed by Liang (1994), which includes 3 items. The questionnaire utilized a 5-point Likert scale, rated from 1 (very poor) to 5 (excellent). Previous studies have shown that this scale has high reliability and validity.

Feelings of inferiority were measured using a scale developed by Wang (1999), consisting of 36 items divided into five dimensions: self-esteem, social confidence, learning ability, appearance, and physical fitness. The questionnaire employed a 5-point Likert scale, rated from 1 (never) to 5 (always). Previous research has demonstrated high reliability and validity for this scale.

Social support was assessed using a scale developed by Chen et al. (2016), which includes 12 items across 3 dimensions: family, friends, and others. The questionnaire used a 5-point Likert scale, rated from 1 (completely disagree) to 5 (completely agree). This scale has also shown high reliability and validity in previous studies.

Emotional regulation ability was measured using a scale developed by Wang et al. (2007), consisting of 14 items divided into two dimensions: expressive suppression and cognitive reappraisal. The questionnaire utilized a 7-point Likert scale, rated from 1 (strongly disagree) to 7 (strongly agree). Previous studies have indicated high reliability and validity for this scale.

Table 3 summarizes the scale indicators used in the study.

**Table 3:** Scales used in this study.

Scale	Author (Year)	Item quantity	Scoring	Dimensions	*M*	SD	α
Physical exercise	Deqing Liang (1994)	3	5	Exercise intensit, Duratio, exercise Frequency	28.97	31.97	0.885
Social support	Qianjin Jiang (2001)	12	5	Family, Friends, Others	3.41	0.65	0.887
Emotional regulation ability	Li Wang (2007)	14	7	Expressive suppression, Cognitive reappraisal	4.46	0.92	0.933
Feelings of inferiority	Lei Wang (1999)	36	5	Self-esteem, Social confidence, Learning ability, Appearance, Physical fitness	3.30	0.66	0.963

### 3.3 Data analysis procedure

The data analysis was conducted following a structured sequence to ensure the reliability and validity of the results. Each step addressed specific aspects of the model and hypotheses, providing a comprehensive understanding of the relationships among physical exercise, social support, emotional regulation ability, and feelings of inferiority.

#### 3.3.1 Descriptive statistics

Initially, descriptive statistics were calculated for all key variables, including physical exercise, social support, emotional regulation ability, and feelings of inferiority. This step provided a foundational understanding of the distribution, mean, and standard deviation of each variable, offering insight into the general characteristics of the sample population.

#### 3.3.2 Common method bias

To assess the potential for common method bias, Harman’s single-factor test was conducted using principal component analysis. The results showed that the first factor accounted for less than 40% of the total variance, suggesting that common method bias was not a significant concern in this study.

#### 3.3.3 Correlation analysis

Correlation analysis was performed to examine the relationships between the key variables. This analysis provided preliminary evidence supporting the hypothesized relationships, showing significant correlations between physical exercise, social support, emotional regulation ability, and feelings of inferiority. The strength and direction of these correlations informed the subsequent SEM analysis.

#### 3.3.4 Differences in key variables across demographic backgrounds

To explore demographic differences, independent sample *t*-tests and one-way ANOVA were conducted. These tests examined whether variables such as physical exercise, social support, emotional regulation ability, and feelings of inferiority differed across demographic factors, including gender and academic year. This step provided insight into whether demographic characteristics influenced the main variables in the study.

#### 3.3.5 Model fit evaluation

Before testing the hypothesized paths, the structural model’s fit was evaluated using multiple fit indices: chi-square to degrees of freedom ratio (χ^2^/df), Comparative Fit Index (CFI), Tucker-Lewis Index (TLI), Standardized Root Mean Square Residual (SRMR), and Root Mean Square Error of Approximation (RMSEA). The model demonstrated an acceptable fit, confirming that the structural equation model (SEM) adequately represented the data structure.

#### 3.3.6 Path analysis

Path analysis was conducted within the SEM framework to test the direct and indirect effects among the variables. The direct effects of physical exercise on feelings of inferiority, social support, and emotional regulation ability were examined, along with the mediating roles of social support and emotional regulation ability in the relationship between physical exercise and feelings of inferiority.

#### 3.3.7 Effect size testing

To evaluate the strength of the direct and indirect effects, effect sizes were calculated using standardized coefficients. Bootstrapping methods were applied to obtain bias-corrected confidence intervals for these effects, providing robust estimates of the mediation pathways. This step ensured that the indirect effects were statistically significant, confirming the mediating roles of social support and emotional regulation ability.

#### 3.3.8 Structural invariance testing across gender

Finally, structural invariance testing was conducted to assess whether the model was consistent across genders. This involved comparing an unconstrained model with progressively constrained models for measurement weights, structural weights, structural covariances, and structural residuals. Minimal changes in fit indices indicated that the model’s structure was invariant across genders, supporting the applicability of the model to both male and female students.

## 4 Results

### 4.1 Common method bias

To evaluate potential common method bias, we conducted Harman’s single-factor test using principal component analysis. The results indicated that the first factor accounted for 33.56% of the total variance, which is below the threshold of 40%. This finding suggests that common method bias is not a significant concern in this study, as no single factor explains a disproportionate amount of the variance.

### 4.2 Correlation analysis among key variables

**Figure 2:**
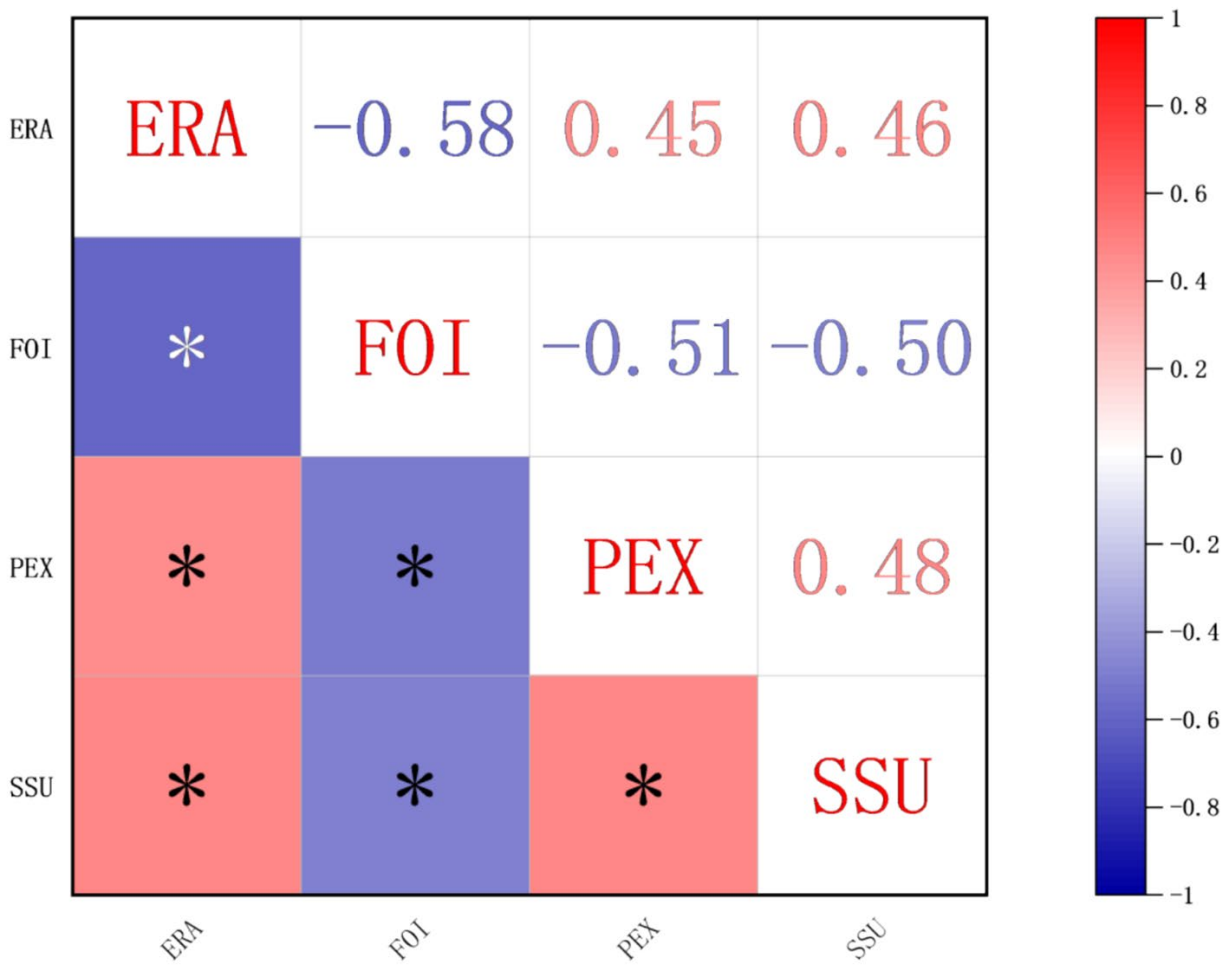
Gender differences in key variables among college students. PEX, Physical exercise. SSU, Social support. ERA, Emotional regulation ability. FOI, Feelings of Inferiority.

Figure 2 presents the correlations among the key variables: physical exercise (PEX), social support (SSU), emotional regulation ability (ERA), and feelings of inferiority (FOI). Significant correlations are marked with an asterisk (*), indicating statistical significance at *p* < 0.05.

Positive correlations: Physical exercise shows significant positive correlations with social support (*r* = 0.48, *) and emotional regulation ability (*r* = 0.45, *), suggesting that students who engage more in physical exercise tend to have higher levels of social support and emotional regulation.

Negative correlations: Feelings of inferiority is significantly negatively correlated with physical exercise (*r* = −0.51, *), social support (*r* = −0.50, *), and emotional regulation ability (*r* = −0.58, *). This indicates that students with higher levels of physical exercise, social support, and emotional regulation report lower levels of inferiority.

In summary, Figure 2 demonstrates that higher levels of physical exercise, social support, and emotional regulation ability are associated with significantly lower levels of feelings of inferiority, underscoring the protective roles of these factors.

### 4.3 Differences in key variables among demographic groups of college students

#### 4.3.1 Gender differences in key variables

To analyze gender differences in key variables among college students, independent sample t-tests were conducted, with the results presented in Table 4. The variables analyzed include physical exercise, social support, emotional regulation ability, and feelings of inferiority.

**Table 4:** Gender differences in key variables among college students.

Variables	Gender	M	SD	t	*P*
Physical exercise	Male	32.17	33.60	4.51	0.000
	Female	25.81	29.96		
Social support	Male	3.35	0.63	−4.63	0.000
	Female	3.48	0.67		
Emotional regulation ability	Male	4.48	0.91	0.68	0.499
	Female	4.45	0.93		
Feelings of inferiority	Male	3.28	0.66	−0.85	0.398
	Female	3.31	0.66		

Physical Exercise: There is a significant difference in physical exercise between male and female students. Male students had a higher mean score (*M* = 32.17, SD = 33.60) compared to female students (*M* = 25.81, SD = 29.96), with a t-value of 4.51 and a *p*-value of 0.000. This indicates that male students tend to engage more in physical exercise than female students.

Social Support: A significant gender difference was also found in social support, where females reported a slightly higher mean (M = 3.48, SD = 0.67) than males (M = 3.35, SD = 0.63), with a t-value of −4.63 and a *p*-value of 0.000. This suggests that female students perceive slightly more social support than their male counterparts.

Emotional Regulation Ability: There was no significant difference between genders in emotional regulation ability. Male students had a mean score of *M* = 4.48 (SD = 0.91), and females had *M* = 4.45 (SD = 0.93), with a t-value of 0.68 and a *p*-value of 0.499, suggesting similar levels across genders.

Feelings of Inferiority: No significant difference was found in feelings of inferiority between genders. The mean scores were similar, with males at *M* = 3.28 (SD = 0.66) and females at *M* = 3.31 (SD = 0.66). The *t*-value was −0.85, with a *p*-value of 0.398, indicating that gender does not have a significant impact on feelings of inferiority.

In summary, Table 4 highlights that gender differences are significant in physical exercise and social support, with males engaging more in physical exercise and females reporting slightly higher social support. However, no significant gender differences were observed in emotional regulation ability or feelings of inferiority.

#### 4.3.2 Grade differences in key variables

To explore differences across grade levels, one-way ANOVA tests were conducted, and the results are presented in Table 5. The variables analyzed include physical exercise, social support, emotional regulation ability, and feelings of inferiority across four grade levels: Freshman, Sophomore, Junior, and Senior.

**Table 5:** Grade differences in key variables among college students.

Variables	Grade	*M*	SD	*F*	*P*
Physical exercise	Freshman (Year 1)	40.90	35.38	56.93	0.000
	Sophomore (Year 2)	31.54	33.18		
	Junior (Year 3)	27.70	30.59		
	Senior (Year 4)	16.24	22.51		
Social support	Freshman (Year 1)	3.28	0.66	12.97	0.000
	Sophomore (Year 2)	3.41	0.65		
	Junior (Year 3)	3.44	0.67		
	Senior (Year 4)	3.52	0.61		
Emotional regulation ability	Freshman (Year 1)	4.22	0.87	28.65	0.000
	Sophomore (Year 2)	4.37	0.93		
	Junior (Year 3)	4.55	0.95		
	Senior (Year 4)	4.71	0.88		
Feelings of Inferiority	Freshman (Year 1)	3.43	0.67	12.74	0.000
	Sophomore (Year 2)	3.31	0.68		
	Junior (Year 3)	3.26	0.66		
	Senior (Year 4)	3.18	0.61		

Physical Exercise: Significant differences in physical exercise were observed across grade levels. Freshmen had the highest mean (*M* = 40.90, SD = 35.38), followed by sophomores (*M* = 31.54, SD = 33.18), juniors (*M* = 27.70, SD = 30.59), and seniors (*M* = 16.24, SD = 22.51), with an *F*-value of 56.93 and a *p*-value of 0.000. This indicates that physical exercise tends to decrease as students progress through their college years.

Social Support: Social support also showed significant differences across grade levels. Seniors reported the highest level of social support (*M* = 3.52, SD = 0.61), while freshmen reported the lowest (*M* = 3.28, SD = 0.66). The ANOVA result was significant, with an *F*-value of 12.97 and a *p*-value of 0.000, suggesting an increase in perceived social support as students advance in grade.

Emotional Regulation Ability: Significant differences were found in emotional regulation ability across grade levels. Seniors had the highest mean (*M* = 4.71, SD = 0.88), while freshmen had the lowest (*M* = 4.22, SD = 0.87). The ANOVA yielded an *F*-value of 28.65 and a p-value of 0.000, indicating that emotional regulation ability increases with grade level.

Feelings of Inferiority: For feelings of inferiority, there were significant differences across grades. Freshmen reported the highest levels (*M* = 3.43, SD = 0.67), while seniors reported the lowest (*M* = 3.18, SD = 0.61), with an *F*-value of 12.74 and a *p*-value of 0.000. This suggests that feelings of inferiority decrease as students progress in their college education.

In summary, Table 5 reveals significant differences across grade levels in all key variables. Physical exercise decreases with each successive year, while social support, emotional regulation ability, and feelings of inferiority show trends where support and regulation ability increase, and inferiority decreases, as students advance in their college years.

### 4.4 Test results of mediation effects

To assess the mediation effects within the model, we first evaluated the model fit using several key indicators, as shown in Table 6. These indicators reflect the adequacy of the questionnaire model in representing the data structure.

**Table 6:** Questionnaire model fitting indicators.

Model fit	χ^2^/*df*	CFI	TLI	SRMR	RMSEA (90%CI)
Model	4.376	0.986	0.980	0.027	0.041 (0.035–0.047)

CFI, comparative fit index; TLI, Tucker-Lewis index. SRMR, standardized root mean square residual. RMSEA, root mean square error of approximation.

Table 6 demonstrates that the model achieves a satisfactory fit across all indicators: χ^2^/df = 4.376, which is below the threshold of 5, indicating an acceptable model fit. CFI = 0.986, which exceeds the recommended threshold of 0.95, suggesting a good fit. TLI = 0.980, also above 0.95, indicating strong model fit. SRMR = 0.027, below the cutoff of 0.08, supporting a good fit. RMSEA = 0.041, with a 90% confidence interval of 0.035 to 0.047, which is well within the acceptable range (typically below 0.08). These results confirm that the model fits the data well, providing a solid foundation for further mediation analysis.

**Figure 3:**
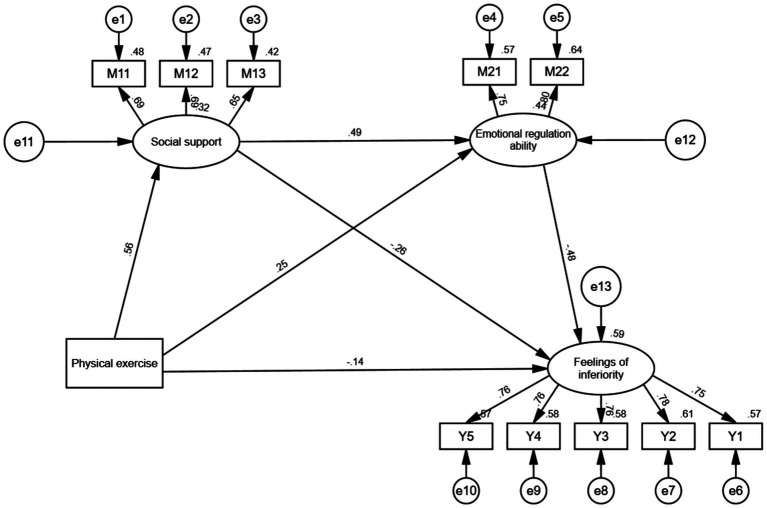
Structural model of the chain mediation effect of social support and emotional regulation ability on the relationship between physical exercise and feelings of inferiority.

Figure 3 presents the structural model evaluating the chain mediation effect of social support and emotional regulation ability on the relationship between physical exercise and feelings of inferiority among college students. The standardized path coefficients in the model help to validate each hypothesis proposed in the study.

Direct Effect of Physical Exercise on Feelings of Inferiority (H1): The path from physical exercise to feelings of inferiority shows a negative direct effect with a standardized coefficient of −0.14. This supports Hypothesis 1 (H1), indicating that increased physical exercise is directly associated with a reduction in feelings of inferiority among college students.

Effect of Physical Exercise on Social Support (H2): Physical exercise has a significant positive effect on social support, with a path coefficient of 0.56. This confirms Hypothesis 2 (H2), suggesting that students who engage in more physical activity experience higher levels of perceived social support.

Effect of Social Support on Feelings of Inferiority (H3): The path from social support to feelings of inferiority is negative and significant, with a coefficient of −0.25. This supports Hypothesis 3 (H3), indicating that higher levels of social support are associated with lower levels of inferiority.

Mediation Effect of Social Support (H4): The indirect effect from physical exercise to feelings of inferiority through social support is confirmed by the model. This pathway suggests that social support partially mediates the relationship between physical exercise and feelings of inferiority, validating Hypothesis 4 (H4). This mediation effect implies that part of the beneficial impact of physical exercise on reducing inferiority operates through enhanced social support.

Effect of Physical Exercise on Emotional Regulation Ability (H5): Physical exercise positively affects emotional regulation ability, as evidenced by the path coefficient of 0.29. This confirms Hypothesis 5 (H5), indicating that students who engage in physical exercise have better emotional regulation skills.

Effect of Emotional Regulation Ability on Feelings of Inferiority (H6): Emotional regulation ability has a strong negative effect on feelings of inferiority, with a coefficient of −0.59. This result supports Hypothesis 6 (H6), suggesting that students with better emotional regulation report significantly lower levels of inferiority.

Mediation Effect of Emotional Regulation Ability (H7): The model shows an indirect effect from physical exercise to feelings of inferiority through emotional regulation ability, confirming Hypothesis 7 (H7). This mediation suggests that the improvement in emotional regulation due to physical exercise contributes to reducing feelings of inferiority among students.

Chain Mediation Effect through Social Support and Emotional Regulation Ability (H8): Finally, the chain mediation pathway from physical exercise to feelings of inferiority through both social support and emotional regulation ability is significant. Physical exercise initially enhances social support (path coefficient = 0.56), which then positively impacts emotional regulation ability (path coefficient = 0.49). This enhanced emotional regulation ability subsequently reduces feelings of inferiority (path coefficient = −0.59), thus confirming Hypothesis 8 (H8). This chain mediation effect highlights the complex interplay of social and emotional resources as mediators in the relationship between physical exercise and feelings of inferiority.

In summary, Figure 3 successfully validates all the proposed hypotheses, providing a comprehensive understanding of how physical exercise impacts feelings of inferiority both directly and indirectly through social support and emotional regulation ability. The direct path confirms the immediate benefits of physical exercise on reducing inferiority, while the indirect paths illustrate how social and emotional resources contribute additional mediating effects. The chain mediation pathway underscores the sequential role of social support in enhancing emotional regulation, which collectively buffers against feelings of inferiority.

Table 7 summarizes the total, direct, and indirect effects in the multiple mediator model, assessing the pathways from physical exercise (PEX) to feelings of inferiority (FOI) through social support (SSU) and emotional regulation ability (ERA). This table provides estimated effects, standard errors (Boot SE), *p*-values, and confidence intervals (Boot LLCI and Boot ULCI), calculated using the Percentile Bootstrap Method with Bias Correction.

**Table 7:** Total, direct and indirect effects in the multiple mediator model.

Path	Estimated effect	Boot SE	P	Boot LLCI	Boot ULCI	Ratio
Direct effect						
PEX → FOI	−0.141	0.025	0.001	−0.189	−0.091	
Indirect effects						
PEX → SSU → FOI	−0.144	0.021	0.001	−0.187	−0.104	26.72%
PEX → ERA→FOI	−0.120	0.017	0.001	−0.155	−0.087	22.26%
PEX → SSU → ERA→FOI	−0.134	0.014	0.000	−0.166	−0.112	24.86%
Total effect	−0.539	0.017	0.001	−0.570	−0.506	

PEX, physical exercise; SSU, social support; ERA, emotional regulation ability; FOI, feelings of inferiority; Boot LLCI, the lower bound of the 95% confidence interval; Boot ULCI, the upper limit of the 95% confidence interval (Percentile Bootstrap Method with Bias Correction). The Bootstrap sample size is set at 2000.

Direct Effect: The direct effect of physical exercise on feelings of inferiority (PEX → FOI) is estimated at −0.141 (Boot SE = 0.025, *p* = 0.001), with a 95% confidence interval of [−0.189, −0.091]. This indicates that physical exercise has a significant direct negative effect on feelings of inferiority, suggesting that higher levels of physical exercise are associated with lower levels of inferiority, even without considering the mediating variables.

Indirect Effects: PEX → SSU → FOI: The indirect effect of physical exercise on feelings of inferiority through social support is −0.144 (Boot SE = 0.021, *p* = 0.001), with a 95% confidence interval of [−0.187, −0.104]. This pathway accounts for 26.72% of the total effect, indicating that social support partially mediates the effect of physical exercise on feelings of inferiority.

PEX → ERA → FOI: The indirect effect of physical exercise on feelings of inferiority through emotional regulation ability is −0.120 (Boot SE = 0.017, *p* = 0.001), with a 95% confidence interval of [−0.155, −0.087]. This pathway contributes 22.26% of the total effect, suggesting that emotional regulation ability also serves as a mediator between physical exercise and feelings of inferiority.

PEX → SSU → ERA → FOI: The chain mediation pathway (physical exercise → social support → emotional regulation ability → feelings of inferiority) shows an indirect effect of −0.134 (Boot SE = 0.014, *p* = 0.000), with a 95% confidence interval of [−0.166, −0.112]. This pathway contributes 24.86% of the total effect, demonstrating the combined mediating roles of both social support and emotional regulation ability.

Total Effect: The total effect of physical exercise on feelings of inferiority (sum of direct and indirect effects) is −0.539 (Boot SE = 0.017, p = 0.001), with a 95% confidence interval of [−0.570, −0.506]. This significant total effect suggests that physical exercise has a strong overall negative association with feelings of inferiority, mediated through social support and emotional regulation ability.

In summary, Table 7 shows that the relationship between physical exercise and feelings of inferiority is significantly mediated by social support and emotional regulation ability. The largest proportion of the total effect is explained by the indirect pathway through social support, followed by the chain mediation effect and the pathway through emotional regulation ability. These findings highlight the complex mechanisms by which physical exercise can reduce feelings of inferiority, with both social and emotional resources playing critical mediating roles.

### 4.5 Testing for structural invariance across gender

To examine whether the structural model is consistent across genders, a test for structural invariance was conducted. Table 8 provides the fit indices for each model with progressively increasing constraints, including the unconstrained model, measurement weights, structural weights, structural covariances, and structural residuals. The indices examined include χ^2^/df, CFI, TLI, SRMR, and RMSEA, as well as changes in CFI (△CFI) and TLI (△TLI) values to assess the model’s invariance across genders.

1. Unconstrained Model: The unconstrained model, which allows parameters to vary freely between genders, shows good fit indices with χ^2^/df = 2.625, CFI = 0.985, TLI = 0.981, SRMR = 0.031, and RMSEA = 0.028 (90% CI, 0.024–0.033). These values suggest an acceptable baseline model fit.
2. Measurement Weights: When constraining the measurement weights to be equal across genders, the model fit remains stable, with only minor changes: χ^2^/df = 2.495, CFI = 0.986 (△CFI = 0.001), TLI = 0.982 (△TLI = 0.001), SRMR = 0.034, and RMSEA = 0.027 (90% CI: 0.023–0.032). This minimal change in fit indicates that the measurement weights are invariant across genders.
3. Structural Weights: Adding constraints on the structural weights yields a χ^2^/df of 2.474, CFI = 0.986 (△CFI = 0.001), TLI = 0.983 (△TLI = 0.002), SRMR = 0.033, and RMSEA = 0.027 (90% CI: 0.023–0.031). The minimal changes in CFI and TLI further support structural weight invariance across genders.
4. Structural Covariances: When the structural covariances are constrained, the model fit indices remain acceptable, with χ^2^/df = 2.592, CFI = 0.984 (△CFI = −0.001), TLI = 0.981 (△TLI = 0), SRMR = 0.030, and RMSEA = 0.028 (90% CI: 0.024–0.032). The slight decrease in CFI (−0.001) is within acceptable limits, indicating that the structural covariances do not vary significantly by gender.
5. Structural Residuals: Finally, constraining the structural residuals yields a χ^2^/df of 2.527, CFI = 0.984 (△CFI = −0.001), TLI = 0.982 (△TLI = 0.001), SRMR = 0.031, and RMSEA = 0.027 (90% CI: 0.023–0.032). This stable fit under structural residual constraints suggests that the residuals are invariant across genders.

**Table 8:** Testing for structural invariance across gender.

	χ^2^/*df*	CFI	△CFI	TLI	△TLI	SRMR	RMSEA (90%CI)
Unconstrained	2.625	0.985	–	0.981	–	0.031	0.028 (0.024–0.033)
Measurement weights	2.495	0.986	0.001	0.982	0.001	0.034	0.027 (0.023–0.032)
Structural weights	2.474	0.986	0.001	0.983	0.002	0.033	0.027 (0.023–0.031)
Structural covariances	2.592	0.984	−0.001	0.981	0	0.030	0.028 (0.024–0.032)
Structural residuals	2.527	0.984	−0.001	0.982	0.001	0.031	0.027 (0.023–0.032)

In summary, Table 8 demonstrates that the model fit remains consistent with each added constraint, and the small changes in CFI and TLI fall within the acceptable range (generally △CFI ≤ 0.01 and △TLI ≤ 0.02). These findings support the structural invariance of the model across genders, indicating that the relationships between physical exercise, social support, emotional regulation ability, and feelings of inferiority are consistent for both male and female students.

## 5 Discussion

### 5.1 The role of physical exercise in reducing feelings of inferiority

The direct negative effect of physical exercise on feelings of inferiority underscores the importance of physical activity in promoting self-worth among young adults. Previous research has linked physical exercise to improved self-esteem, body image (Chen et al., 2006; Ma and Liu, 2015), and mental health, which this study corroborates by showing a direct link to reduced feelings of inferiority. This relationship can be explained by the self-affirming nature of physical exercise. Regular engagement in exercise may foster a sense of competence and achievement, both of which are essential for maintaining a positive self-concept. This finding supports the application of physical exercise as a primary strategy in reducing inferiority feelings, suggesting that even without other interventions, physical activity alone can significantly impact students’ psychological health.

### 5.2 Social support as a mediating mechanism

This study reveals the critical role of social support as a mediating variable, effectively buffering against negative self-perceptions by enhancing students’ sense of connection and belonging. Whether in college settings or other social contexts, team sports or group fitness activities naturally promote social interaction and peer bonding. These social benefits are not limited to specific cultural or educational environments but may also operate in diverse settings, such as community fitness programs or workplace health initiatives.

Furthermore, social support, as a universal resource for mental health, has been widely documented for its role in reducing stress and enhancing self-esteem (Liu and Huang, 2010; Li and Li, 2015). By validating the mediating effect of social support, this study further emphasizes its universality. This suggests that physical activity programs should prioritize social components, such as group activities or team-based exercises, to maximize psychological benefits by fostering peer support networks.

### 5.3 Emotional regulation ability as a critical pathway

Emotional regulation ability emerged as another significant mediator in the relationship between physical exercise and feelings of inferiority. Emotional regulation skills, including the ability to identify, understand, and manage emotions, are essential for alleviating negative self-perceptions (Gross, 2002; Liu et al., 2020). Studies have shown that physical exercise enhances emotional regulation by promoting physiological balance, reducing stress hormones, and fostering a more stable emotional state. This study further highlights how improved emotional regulation through exercise reduces feelings of inferiority.

From a developmental psychology perspective, emotional regulation ability is crucial across all age groups. Whether for college students, early-career professionals, or individuals transitioning into retirement, emotional regulation skills help individuals better cope with life transitions and challenges. This study suggests that physical exercise can play a key role in this developmental process by enhancing students’ emotional resilience. By gaining better control over their emotional responses, students become less vulnerable to feelings of inadequacy and better equipped to maintain a positive self-image. These findings underscore the importance of incorporating physical activity interventions targeting emotional skills into educational programs. Improved emotional regulation not only enhances mental health but also strengthens resilience against feelings of inferiority and related psychological challenges.

### 5.4 The chain mediation effect of social and emotional factors

The chain mediation effect, where physical exercise influences feelings of inferiority sequentially through social support and emotional regulation ability, provides a novel insight into the interconnected nature of social and emotional resources. This pathway emphasizes that the benefits of physical exercise are not limited to isolated psychological effects but are, in fact, a cascade of positive outcomes. Social support, as the initial mediator, fosters a foundation of connectedness and belonging, which subsequently strengthens emotional regulation abilities. This layered mechanism suggests that social experiences derived from physical exercise may create a secure base that enhances emotional self-regulation, leading to a holistic improvement in students’ psychological resilience.

This finding is consistent with ecological models of development, which posit that individuals function within interconnected systems (Liu and Huang, 2010). The current study’s model supports this view, suggesting that social and emotional systems work in tandem to influence individual mental health outcomes. Physical exercise, therefore, not only addresses the individual’s physical and psychological health but also reinforces the social and emotional networks that buffer against negative self-perceptions. This chain effect implies that interventions should focus on creating environments where social support and emotional skill-building are integrated with physical activities to maximize overall well-being.

### 5.5 Gender consistency in the structural model

The invariance testing across gender reveals that the pathways from physical exercise to social support, emotional regulation ability, and ultimately, feelings of inferiority are consistent for both male and female students. This structural consistency suggests that the benefits of physical exercise and its mediating mechanisms are robust across genders, supporting the universality of physical exercise as an intervention strategy. This finding has important implications for designing inclusive exercise programs on college campuses, where gender differences in participation and perception of physical activities may exist. By showing that both male and female students benefit equally from the exercise-social–emotional model, the study advocates for campus-wide, gender-neutral physical activity interventions.

### 5.6 Implications for intervention and policy

The findings of this study have several practical implications for mental health interventions and policy-making within educational settings. First, integrating structured physical exercise programs into college mental health initiatives could be a highly effective approach to improving students’ psychological resilience. Institutions should consider offering group-based exercise programs that facilitate social interaction, as this enhances both social support and emotional regulation abilities, contributing to lower levels of inferiority. Furthermore, counseling services could collaborate with fitness and recreation departments to develop workshops that combine physical exercise with emotional regulation training, targeting students’ comprehensive well-being.

From a policy perspective, college administrations should recognize the role of physical activity in mental health and allocate resources toward developing accessible exercise programs. Promoting awareness of the psychological benefits of exercise and removing barriers to participation could foster a more supportive and mentally healthy campus environment. The multi-level impacts of physical exercise identified in this study underline the need for policies that view physical health, social networks, and emotional well-being as interconnected aspects of students’ lives, requiring a holistic approach.

### 5.7 Limitations and future directions

Although this study provides valuable insights into the relationships among physical exercise, social support, emotional regulation, and feelings of inferiority, there is room for improvement, which future research could further refine.

First, while the cross-sectional design reveals associations among variables, it has certain limitations in inferring causal relationships. Future studies could employ longitudinal designs to monitor the dynamic changes among variables, thereby offering a more comprehensive understanding of their causal pathways and long-term impacts.

Second, this study primarily relies on self-reported data for information collection. While this method is practical and widely applicable, it may be influenced by response biases (e.g., social desirability bias or recall errors), potentially compromising the objectivity of the data. Future research could integrate more objective measurement methods, such as using wearable devices to track physical activity data or employing observational and behavioral assessments to evaluate social support and emotional regulation. These approaches would further enhance the reliability and validity of the findings.

Moreover, although the variables examined in this study cover key psychological and social dimensions, other important factors that may influence feelings of inferiority remain underexplored. For instance, psychological constructs such as self-efficacy and resilience might act as mediators or moderators in the relationship between physical exercise and mental health. Future research could incorporate these variables into analytical models to explore their potential complex mechanisms, providing a more detailed theoretical foundation for understanding the psychological benefits of physical exercise.

In summary, future research directions could focus on the following areas: adopting longitudinal designs to better elucidate causal relationships, integrating diverse and objective measurement methods to improve data accuracy, including more potential mediating or moderating variables to expand the theoretical framework, and broadening the cultural and demographic diversity of samples to enhance the generalizability of the findings. These improvements will help deepen the understanding of the psychological benefits of physical exercise and provide a robust theoretical basis for developing more targeted intervention strategies.

## 6 Conclusion

In summary, this study advances our understanding of the mechanisms by which physical exercise influences mental health among college students, identifying social support and emotional regulation as key mediating factors. The findings suggest that physical exercise not only directly reduces feelings of inferiority but also strengthens social and emotional resources that further contribute to mental resilience. These insights highlight the importance of holistic intervention strategies that incorporate physical, social, and emotional dimensions, emphasizing the value of physical exercise as a foundational component of college mental health programs.

## Data Availability

The original contributions presented in the study are included in the article/Supplementary material, further inquiries can be directed to the corresponding author.
