# Supplementary material for: A study on the relationship between physical exercise and feelings of inferiority among college students: the chain mediating effect of social support and emotional regulation ability
Source: Front Psychol. 2025 Jan 9;15:1521510. doi: 10.3389/fpsyg.2024.1521510 (PMC11754417; doi:10.3389/fpsyg.2024.1521510)
Supplement: Supplementary file 1 [file Data_Sheet_1.ZIP › S1 Empirical data and original scale/S1 original scale.pdf]

Questionnaire on the relationship between physical exercise and feelings of inferiority in university students: the chain mediation effect of social Support and emotional regulation ability

Dear Student,

Hello! Thank you for taking the time to participate in this questionnaire. This survey aims to explore the relationship between physical exercise and feelings of inferiority among university students, with a focus on the chain mediation effects of social support and emotional regulation ability. The objective of this study is to investigate the interactions among these factors, providing a scientific basis to improve mental health and physical fitness among university students.

The survey primarily consists of objective questions. Please respond according to your actual experiences. Given that individual circumstances vary, there are no right or wrong answers for each question in this survey. Furthermore, all data collected in this research will be kept strictly confidential and will not be disclosed publicly, nor will any personal evaluations be made. Please feel comfortable answering truthfully. Select the option that best represents your situation.

I solemnly pledge to maintain strict confidentiality regarding all information you provide.

Your cooperation and support are essential to this research, and I am very grateful for your assistance!

Part 1: Personal Information (Please mark "√" next to the appropriate option)

1. Gender: Male ( ) Female ( )

2. Grade: First Year ( ) Second Year ( ) Third Year ( ) Fourth Year ( )

3. Home Address: Urban ( ) Rural ( )

Part 2: Physical Exercise Level Scale

Please recall your physical exercise over the past month and select the corresponding answer (A, B, C, D, E) in the parentheses based on your actual situation.

1. **What type of physical exercise do you regularly engage in?** ( )

A. Light exercise (e.g., walking, doing light calisthenics)

B. Low-intensity, relaxed exercise (e.g., casual volleyball, table tennis, jogging, tai chi)

C. Moderate-intensity, more vigorous and enduring exercise (e.g., cycling, running)

D. High-intensity exercise with heavy breathing and sweating, but not very prolonged (e.g., badminton, basketball, tennis, soccer)

E. High-intensity and prolonged exercise with heavy breathing and sweating (e.g., racing, aerobic routines, swimming)

2. When engaging in the above intensity of physical activities, how many minutes do you usually spend each time? ( )

A. Less than 10 minutes

B. 11 to 20 minutes

C. 21 to 30 minutes

D. 31 to 59 minutes

E. 60 minutes or more

3. How many times per month do you engage in the above physical activities? ( )

A. Less than once per month

B. 2 to 3 times per month

C. 1 to 2 times per week

D. 3 to 5 times per week

E. Approximately once per day

Feelings of Inferiority Scale

**Serial number**	**Item**	**①Never ②Rarely ③Sometimes ④Often ⑤Always**				
1	Do you often feel that you are inferior to most people you know?	1	2	3	4	5
2	Have you ever thought of yourself as a worthless person?	1	2	3	4	5
3	How confident are you that people you know will someday respect and appreciate you?	1	2	3	4	5
4	Have you ever felt so discouraged about yourself that you began to question your own worth?	1	2	3	4	5
5	Do you often feel dislike towards yourself?	1	2	3	4	5

6	Generally speaking, how confident are you in your abilities?	1	2	3	4	5
7	Do you often feel that you can't do anything right?	1	2	3	4	5
8	How much do you worry about getting along with other people?	1	2	3	4	5
9	Do you often worry about doing something that might invite criticism from teachers or employers?	1	2	3	4	5
10	When you walk into a room where people are gathered and talking, have you ever felt fear and anxiety?	1	2	3	4	5
11	Do you often feel uncomfortable?	1	2	3	4	5
12	How much do you worry about whether others view you as a success or a failure in your work or studies?	1	2	3	4	5
13	Do you find it difficult to come up with appropriate topics of conversation when in a crowd?	1	2	3	4	5
14	When you make an embarrassing mistake or do something that makes you look foolish, how long does it take you to forget it?	1	2	3	4	5
15	Do you often feel uncomfortable when meeting strangers?	1	2	3	4	5
16	Do you often worry about whether people will want to be around you?	1	2	3	4	5
17	Are you often troubled by shyness?	1	2	3	4	5

18	When you think that people you meet have a poor opinion of you, how concerned or worried are you about it?	1	2	3	4	5
19	Do you often feel anxious or uneasy about how others perceive you?	1	2	3	4	5
20	If you need to read a passage and understand its meaning in class, how worried or anxious are you about it?	1	2	3	4	5
21	When you have to write a persuasive piece for a teacher who may disagree with you, how worried or anxious are you?	1	2	3	4	5
22	Do you often feel difficulty in assignments that require you to express your views in writing?	1	2	3	4	5
23	How frequently do you encounter difficulties in reading comprehension exercises in class?	1	2	3	4	5
24	Do you often imagine that your learning ability is inferior to your classmates?	1	2	3	4	5
25	How often do you feel confident about doing well on important assignments, like a term paper?	1	2	3	4	5
26	Compared to your classmates, how often do you feel you need to study harder to achieve the same results?	1	2	3	4	5
27	Have you ever felt ashamed of your physique or	1	2	3	4	5

	appearance?					
28	Do you often feel that most of your friends or peers are more attractive than you physically?	1	2	3	4	5
29	Do you often wish or fantasize about becoming more attractive?	1	2	3	4	5
30	Have you ever felt worried or anxious about your ability to attract the opposite sex?	1	2	3	4	5
31	How confident are you that others find your appearance attractive?	1	2	3	4	5
32	Have you ever thought of yourself as physically uncoordinated?	1	2	3	4	5
33	Do you feel that your athletic abilities are inferior to most others?	1	2	3	4	5
34	When you participate in sports activities that require coordination, do you often worry about not doing well?	1	2	3	4	5
35	Have you ever thought you lack talent in dancing or other coordination-based physical activities?	1	2	3	4	5
36	When you try hard to perform well in a sport and know others are watching, do you feel nervous or uneasy?	1	2	3	4	5

Social Support Scale

**Serial number**	**Item**	**①Completely Disagree**

		②Disagree<br>③Somewhat Agree<br>④Agree<br>⑤Completely Agree				
1	My family can provide me with tangible and practical help.	1	2	3	4	5
2	When I need it, I can receive emotional help and support from my family.	1	2	3	4	5
3	I can discuss my problems with my family.	1	2	3	4	5
4	My family is willing to help me make various decisions.	1	2	3	4	5
5	My friends can genuinely help me.	1	2	3	4	5
6	In times of difficulty, I can rely on my friends.	1	2	3	4	5
7	My friends can share both happiness and sorrow with me.	1	2	3	4	5
8	I can discuss my problems with my friends.	1	2	3	4	5
9	When I encounter problems, some people (teachers, classmates) will be there for me.	1	2	3	4	5
10	I can share both happiness and sorrow with certain people (teachers, classmates).	1	2	3	4	5
11	When I am in trouble, some people	1	2	3	4	5

	(teachers, classmates) are a true source of comfort for me.					
12	Certain people (teachers, classmates) in my life care about my feelings.	1	2	3	4	5

Emotional Regulation Scale

Serial number	Item	①Strongly Disagree ②Disagree ③Somewhat Disagree ④Neutral ⑤Somewhat Agree ⑥Agree ⑦Strongly Agree						
1	When I feel happy, I try not to show it.	1	2	3	4	5	6	7
2	When I feel sad, I suppress this emotion and don’t let others know my true feelings.	1	2	3	4	5	6	7
3	I don’t show my emotions.	1	2	3	4	5	6	7
4	When I feel fear, I don’t let my emotions show.	1	2	3	4	5	6	7
5	When I feel angry, others won’t know from my appearance that I am truly angry inside.	1	2	3	4	5	6	7
6	I control my emotions by not expressing them.	1	2	3	4	5	6	7
7	When I dislike someone or something, I suppress this feeling and don’t let it show.	1	2	3	4	5	6	7

8	When faced with a situation that would make me angry, I change the way I view the problem to lessen my anger.	1	2	3	4	5	6	7
9	I change the way I think about a situation to reduce my feelings of dislike towards someone or something.	1	2	3	4	5	6	7
10	I change the way I understand a situation to control my emotions.	1	2	3	4	5	6	7
11	When faced with a situation that would make me sad, I think about it from a different perspective to lessen my sadness.	1	2	3	4	5	6	7
12	I try to change my view of my surroundings to make myself feel happier.	1	2	3	4	5	6	7
13	When faced with a situation that would make me fearful, I change the way I view the situation to reduce my fear.	1	2	3	4	5	6	7
14	I change the way I think about things to regulate my emotions.	1	2	3	4	5	6	7

The questionnaire is now complete. Thank you for your responses!
